# Cardiovascular risk factor treatment targets and renal complications in high risk vascular patients: a cohort study

**DOI:** 10.1186/1471-2261-11-40

**Published:** 2011-07-05

**Authors:** Sharmini Selvarajah, Yolanda vD Graaf, Frank LJ Visseren, Michiel L Bots

**Affiliations:** 1 Clinical Research Centre, Kuala Lumpur Hospital, Kuala Lumpur, Malaysia; 2 Julius Center for Health Sciences and Primary Care, University Medical Center Utrecht, Utrecht, The Netherlands; 3 Department of Vascular Medicine, University Medical Center Utrecht, Utrecht, The Netherlands

## Abstract

**Background:**

To determine if recommended treatment targets, as specified in clinical practice guidelines for the management of cardiovascular disease, reduces the risk of renal complications in high risk patient populations.

**Methods:**

This was a cohort study. Participants in Utrecht, The Netherlands either at risk of, or had cardiovascular disease were recruited. Cardiovascular treatment targets were achievement of control in systolic and diastolic blood pressure, total and low-density cholesterol, and treatment of albuminuria. Outcome measures were time to development of end stage renal failure or symptomatic renal atherosclerotic disease requiring intervention.

**Results:**

The cohort consisted of 7,208 participants; 1,759 diabetics and 4,859 with clinically manifest vascular disease. The median age was 57 years and 67% were male. Overall, 29% of the cohort achieved the treatment target for systolic blood pressure, 39% for diastolic blood pressure, 28% for total cholesterol, 31% for LDL cholesterol and 78% for albuminuria. The incidence rate for end stage renal failure and renal atherosclerotic disease reduced linearly with each additional treatment target achieved (p value less than 0.001). Achievement of any two treatment targets reduced the risk of renal complications, hazard ratio 0.46 (95% CI 0.26-0.82). For patients with clinically manifest vascular disease and diabetes, the hazard ratios were 0.56 (95% CI 0.28 - 1.12) and 0.28 (95%CI 0.10 - 0.79) respectively.

**Conclusion:**

Clinical guidelines for cardiovascular disease management do reduce risk of renal complications in high risk patients. Benefits are seen with attainment of any two treatment targets.

## Background

Current clinical practice guidelines for the management of patients with diabetes, hypertension and other atherosclerotic risk factors are geared to the prevention of cardiovascular disease and its complications [1-3]. However, cardiovascular diseases are not the only complications that can arise. Renal complications such as renal atherosclerotic disease [4] and end stage renal failure (ESRF) are of equal importance [5-9] though not as common.

There are few studies looking at the effects of combined cardiovascular treatment targets on renal complications; most are aimed at cardiovascular complications for which the targets were derived for. The risk of renal complications is low [10] despite the high prevalence of diabetes and hypertension [11,12], and is usually confined to those with a genetic predisposition [13,14]. In clinical practice, these complications arise after a long duration of disease and/or treatment [15]. In the meantime, these patients undergo the same clinical management as those who are not predisposed to renal diseases. Studies of natural history of reno-atherosclerotic disease have shown that control of blood pressure do not necessarily prevent the progression of renal disease [16]. Therefore, it is relevant to determine whether the current clinical guidelines which have treatment targets geared for cardiovascular disease reduce the risk of renal complications.

This study will evaluate if the current 2007 European treatment guidelines for the prevention of cardiovascular disease concomitantly reduces the risk of renal complications in patients at high risk of vascular diseases. We assessed whether there is an inverse dose-response risk for renal complications by the number of treatment targets achieved or if there is an optimal number instead. We also determined if there are differences in targets to be achieved for two high risk patient groups; diabetics and those with clinically manifest vascular disease.

## Methods

### Study design and population

The Second Manifestations of ARTerial disease (SMART) study is a prospective cohort study that is conducted in the University Medical Centre (UMC) Utrecht. It commenced in September 1996 and is currently on-going. Patients aged 18 to 80 years with clinically manifest atherosclerotic vascular disease, newly referred to the UMC are invited to participate. Diseases that qualify for enrolment are internal carotid artery stenosis, transient ischemic attack, minor stroke, peripheral arterial disease, diabetic foot, angina pectoris, myocardial infarction, abdominal aortic aneurysm, and renal artery stenosis. Those treated for cardiovascular risk factors, including hyperlipidaemia, diabetes mellitus, hypertension and renal insufficiency are also recruited. Patients with terminal cancer, dependent in their daily activities or not fluent in Dutch are excluded. All cohort members are followed up for a minimum of three years using biannual questionnaires.

The study was approved by the UMC Utrecht Institutional Review Board, which is approved by the Dutch Central Committee for research involving human subjects. The study was conducted in accordance with principles of the Declaration of Helsinki. Written informed consent was obtained from all participants. The rationale and design of the SMART study have been described elsewhere [17].

The current study selected all patients without end stage renal failure at baseline, recruited from September 1996 till February 2008. Every patient who was enrolled in the SMART study had physical examinations at baseline. Blood pressure was measured using a sphygmomanometer on both upper arms. The measurement was repeated on the arm with the highest value. The maximal value was used in this study.

Hypertension was diagnosed based on a systolic blood pressure of more or equal to 140 mm Hg and/or a diastolic blood pressure of more or equal to 90 mm Hg or being treated with two or more anti-hypertensive medications. Diabetes was determined based on self-report, hyperglycaemia at baseline (> 7.0 mmol L^-1^), glycated haemoglobin (HbA1c) of more than 7% or being treated with glucose lowering therapy.

### Clinical measurements

The exposure of interest was number of cardiovascular risk factor treatment targets achieved at baseline. Treatment targets were defined according to the 2007 European guidelines [18] for high risk patients and were limited to the management of risk factors that were modifiable using drug therapy. Targets were systolic and diastolic blood pressure, total cholesterol and low density lipoprotein (LDL) and albuminuria. For albuminuria status, the target to achieve was no micro-albuminuria. For those with albuminuria (micro-albuminuria or proteinuria), the desired target was the appropriate choice of therapy for albuminuria; treatment with either an angiotensin-converting enzyme inhibitor (ACEI) or angiontensin II receptor blocker (ARB). The choice of renin-angiotensin system (RAS) inhibitor use as a treatment goal is due to its effects on albuminuria, independent of blood pressure lowering. RAS inhibitors either improve albuminuria by reducing excretion (regression or stabilization)[19-21] or by delaying the time to progression [22]. Diabetics had an additional target; HbA1c. Patients had a maximum of five targets to achieve whereas those with diabetes had six. Table 1 depicts the treatment targets for this study. In the final analysis, the treatment categories were condensed to five categories; ≤ 1: those who had none or only one target achieved, 2, 3, 4, ≥ 5 those who had five and/or all targets achieved.

**Table 1:** Cardiovascular risk factor treatment targets

	Treatment targets	
Risk factor	All patients without Diabetes	Diabetes mellitus
Systolic blood pressure (mm Hg)	< 130	< 130
Diastolic blood pressure (mm Hg)	< 80	< 80
Total cholesterol (mmol L^-1^)	< 4.5	< 4.5
Low density lipoprotein (mmol L^-1^)	< 2.5	< 2.5
HbA1c (%)	-	< 6.5
Microalbuminuria and proteinuria	Treatment with ACEI/ARB or No proteinuria	Treatment with ACEI/ARB (Any albuminuric state)

ACEI: angiotensin-converting enzyme inhibitor

ARB: angiontensin II receptor blocker

### Biochemical measurements

The biochemical assays measured in this study has been described in detail previously [17]. In summary, a venous blood sample was taken to determine lipid, glucose and creatinine levels. Plasma total cholesterol, triglycerides, glucose and creatinine were measured using a commercial enzymatic dry chemistry kit (Johnson and Johnson). HDL-cholesterol in plasma was determined using a commercial enzymatic kit (Boehringer-Mannheim) after precipitation of LDL and very low density lipoprotein (VLDL) with sodium phosphotungstate magnesium chloride. LDL-cholesterol was calculated using the Friedewald formula.

A urine sample was collected to measure micro-albuminuria and creatinine excretion. Creatinine was measured using a commercial enzymatic dry chemistry kit (Johnson and Johnson) and micro-albuminuria was determined with immunoturbidimetric assays (Boehringer-Mannheim).

### Primary outcome

Renal outcomes were defined as a composite endpoint consisting of end stage renal failure requiring renal replacement therapy and reno-atherosclerotic disease requiring intervention; either arterial stenting or bypass grafting. Time to any renal endpoint was the outcome of interest.

During the follow up period, outcomes were determined through questionnaires biannually. Participants provided information on hospitalization and clinic visits. Original source documents were reviewed if a hospitalization or clinic visit was reported. All hospital discharge letters and results of related laboratory and radiological examinations were collected. Each event was classified according to a standard operating procedure. All endpoints were adjudicated by three members of the SMART Endpoint Committee, comprising of physicians from different departments.

The participants were followed up till death or refusal to participate.

### Statistical analysis

Analysis was performed using SPSS version 17.0 for Windows (SPSS Inc., Chicago, IL, USA). Baseline data displayed was calculated using a general linear model for continuous variables adjusted for age and sex. P values for trends across the five treatment target groups were estimated using Mantel Haenszel's χ2. Ordinal data was tested using ordinal tests (Kendall's tau).

Confounders were selected a priori for testing based on literature. Variables selected were age, sex, waist circumference, history of coronary artery disease, cerebrovascular disease, peripheral arterial disease and renal disease, smoking status (never, former, current), alcohol consumption (never, ever, recently stopped, current), haemoglobin levels, albuminuria status (no, micro-albuminuria, overt proteinuria) and estimated glomerular filtration rate (eGFR) using the Modification of Diet in Renal Disease (MDRD) formula. These were then determined to be included in the final model if there was a change in the hazard ratio (HR) estimate of more than 10 percent when compared to the crude estimate [23]. Medication was seen as not confounders in this study because the type and number of drugs used were primarily to achieve treatment targets. Medications which had independent action on renal function such as ACEIs and ARBs were already included as a treatment target for albuminuria.

A multivariate Cox proportional hazards model was used to determine the association between the number of treatment targets achieved and the risk of renal complications. Hazard ratios and the 95% confidence intervals were calculated for each number of treatment targets achieved, with the category of one or less targets achieved as reference. The likelihood ratio test was used to determine the significance of the variables included in the model. A p value of less than 0.05 (two tailed) was considered to be statistically significant. Analysis was performed with two adjustments; 1) adjustment for statistically significant confounders 2) adjustment for statistically significant confounders, age and sex. For participants from the whole cohort, the significant confounders were hypertension, albuminuria, eGFR and haemoglobin levels. For those with clinically manifest vascular disease, the confounders were hypertension, albuminuria and eGFR levels. Only eGFR and haemoglobin were significant confounders for participants with diabetes. The Wald test was used to determine if treatment targets were significant as a linear variable. A p value of less than 0.05 was considered to be statistically significant.

The assessment of hazard ratios for individual treatment targets was done at a quantitative level. There was a statistically significant correlation between systolic and diastolic blood pressure. Pearson's correlation coefficient *r *was 0.68 with a p value of less than 0.001 (two tailed). There was stronger correlation between total cholesterol and LDL levels, Spearman's correlation coefficient *rho *0.88 was statistically significant at a p value of less than 0.001 (two tailed). When testing the hazard ratios at the risk factor level, due to the high correlation between these variables, only one from each set of these factors was chosen; systolic blood pressure and LDL. Both systolic blood pressure and LDL cholesterol levels are the more important treatment targets for the prevention of cardiovascular disease [24,25]. Cox proportional hazards assumptions were tested visually using hazard and *Log Minus Log *(LML) plots.

Incidence rates were calculated using the number of events, divided by the total person time in years. The time period is calculated from baseline till event, death or loss to follow up.

Missing data was reviewed to determine if it was Missing At Random. Those missing at less than one percent were imputed using mean or median values where applicable. Single imputation using a linear regression with an error term was done for other variables. This was done for four variables; albuminuria (6.2% missing), LDL (6.9% missing), waist circumference (17.6% missing) and HbA1c for diabetics (38.1% missing).

## Results

Between September 1996 and February 2008, 7,292 participants were enrolled in the SMART study cohort. Of these, the 84 participants who had end stage renal failure at baseline were excluded from this study. At the end of the study period, 670 had died and 253 were lost to follow up. The median duration for follow up was 4.21 years (IQR 2.08 - 7.11).

### Characteristics of the participants

The cohort consisted of 7,208 participants. There were 4,859 (67.4%) with clinically manifest vascular disease and 1,759 (24.4%) diabetics, both conditions not mutually exclusive. Other participants had risk factors without clinical evidence of atherosclerotic disease. The median age was 57 years (IQR 48 - 66) and 67.2% were male. Overall, 29.1% of the cohort fulfilled the treatment target for systolic blood pressure, 38.5% for diastolic blood pressure, 28.2% for total cholesterol, 30.7% for LDL cholesterol and 77.9% for albuminuria. About 47% of those prescribed beta blockers had ischaemic heart disease. Only 12% of those prescribed beta blockers were due to hypertension.

Table 2 summarizes the baseline characteristics of the whole cohort. Those who achieved all five targets were more likely to be male, had more often a previous history of coronary artery disease, consumed alcohol, had lower levels of triglycerides and micro-albuminuria or proteinuria was less common.

**Table 2:** Clinical profile and end points of study population according to number of cardiovascular risk factor treatment targets achieved at baseline

	Treatment Targets Achieved					
Characteristics	≤ 1	2	3	4	≥ 5	p value for trends
n	2813	1652	1785	593	365	
Age (years)^a^	57 (49,66)	57 (48,66)	56 (47,64)	58 (50,66)	55 (46,64)	
Male sex	65.6 (1845)	65.9 (1088)	67.3 (1202)	73.9 (438)	75.1 (274)	0.001
Smoking						
*Current*	32.1 (902)	33.7 (556)	30.3 (541)	29.3 (174)	30.7 (112)	
*Former*	41.7 (1174)	42.3 (698)	45.2 (806)	43.8 (260)	46.3 (169)	
*Never*	26.2 (737)	24.1 (398)	24.5 (438)	26.8 (159)	23 (84)	
*Packyears *	18 ± 0.36	19 ± 0.46	18 ± 0.45	18 ± 0.77	17 ± 0.99	
Alcohol consumption						0.001
*Never*	20.8 (584)	21.7 (358)	19.9 (355)	18.2 (108)	18.1 (66)	
*Ever*	9.7 (273)	9 (148)	9.8 (175)	10.8 (64)	12.9 (47)	
*Recently stopped*	29.5 (831)	28.5 (470)	23.1 (412)	16.5 (98)	14.2 (52)	
*Current*	40 (1125)	40.9 (676)	47.2 (843)	54.5 (323)	54.8 (200)	
History of:						
*Coronary artery disease*	26.5 (745)	36.7 (607)	49 (875)	63.4 (376)	71 (259)	0.001
*Cerebrovascular disease*	19.9 (561)	20.8 (343)	17.9 (319)	18.4 (109)	15.6 (57)	0.027
*Peripheral arterial disease*	16.8 (472)	17.1 (283)	13 (232)	8.4 (50)	7.9 (29)	0.001
*Previous kidney disease*	2.6 (74)	2.7 (44)	2.7 (48)	4.9 (29)	3.6 (13)	
*Any of the above*	56.7 (1595)	64.5 (1065)	71.8 (1281)	82.1 (487)	87.1 (318)	0.001
BMI (kg m^-2^)	27 ± 0.08	27 ± 0.11	27 ± 0.10	27 ± 0.18	26 ± 0.23	0.001
Waist circumference (cm)	95 ± 0.22	94 ± 0.29	93 ± 0.28	94 ± 0.49	92 ± 0.62	0.001
Systolic BP (mm Hg)	154 ± 0.34	141 ± 0.44	135 ± 0.43	130 ± 0.74	119 ± 0.95	0.001
Diastolic BP (mm Hg)	91 ± 0.20	82 ± 0.26	79 ± 0.25	75 ± 0.44	71 ± 0.56	0.001
S. Glucose (mmol L^-1^)	6.5 ± 0.04	6.6 ± 0.06	6.1 ± 0.05	6.6 ± 0.09	5.9 ± 0.12	0.001
HbA1c (%)	6.1 ± 0.02	6.2 ± 0.03	5.9 ± 0.03	6.1 ± 0.05	5.8 ± 0.06	0.001
Total cholesterol (mmol L^-1^)	5.95 ± 0.02	5.45 ± 0.03	4.87 ± 0.03	4.02 ± 0.05	3.76 ± 0.06	0.001
HDL cholesterol (mmol L^-1^)	1.28 ± 0.01	1.25 ± 0.01	1.25 ± 0.01	1.24 ± 0.02	1.20 ± 0.02	0.001
LDL cholesterol (mmol L^-1^)	3.75 ± 0.02	3.23 ± 0.03	2.84 ± 0.03	2.04 ± 0.05	1.95 ± 0.06	0.001
Triglycerides (mmol L^-1^)	2.05 ± 0.04	2.16 ± 0.05	1.74 ± 0.05	1.65 ± 0.09	1.38 ± 0.11	0.001
Creatinine (μmol L^-1^)	92.3 ± 0.87	93.9 ± 1.13	92.1 ± 1.09	91.6 ± 1.88	93.1 ± 2.40	
Microalbuminuria	21.9 (616)	16.5 (272)	9.7 (174)	13.8 (82)	7.9 (29)	0.001
Proteinuria	3.6 (102)	2.8 (46)	1.4 (25)	1.9 (11)	0.8 (3)	
eGFR (mL min-1 1.73 m-2)	78.1 ± 0.33	77.6 ± 0.43	77.9 ± 0.41	78.7 ± 0.71	78.1 ± 0.91	
Haemoglobin (mmol L^-1^)	9.0 ± 0.01	8.8 ± 0.02	8.8 ± 0.02	8.7 ± 0.03	8.6 ± 0.04	0.001
Patients with Hypertension	84.9 (2387)	62.3 (1029)	54.2 (968)	55.3 (328)	41.4 (151)	0.001
Duration of hypertension (years)	6.0 ± 0.19	5.1 ± 0.24	4.8 ± 0.23	5.5 ± 0.40	4.5 ± 0.51	0.001
Use of BP lowering drugs	62.2 (1749)	61.8 (1021)	64.8 (1157)	75.9 (450)	79.2 (289)	0.001
Type of BP lowering drugs						
*β-blockers*	29.6 (834)	36 (594)	42.7 (762)	50.9 (302)	60.8 (222)	0.001
*Diuretics*	18.3 (516)	16.6 (274)	16.6 (296)	22.8 (135)	16.4 (60)	
*ACE inhibitors*	20.5 (576)	22.8 (377)	26.8 (478)	33.1 (196)	32.3 (118)	0.001
*ARBs*	7.2 (203)	6.6 (109)	7.1 (126)	11.5 (68)	8.5 (31)	
*Calcium channel blockers*	15.2 (428)	15.7 (260)	16.1 (288)	20.2 (120)	18.1 (66)	0.015
Number of BP lowering drugs						0.001
*1*	27.3 (768)	26.8 (443)	26.3 (470)	28.8 (171)	37.5 (137)	
*2*	18.3 (515)	18.9 (313)	22.7 (406)	27 (160)	24.7 (90)	
*3*	7.6 (214)	9.1 (151)	10.2 (182)	13.8 (82)	14.8 (54)	
≥ *4*	1.8 (51)	2.1 (33)	2.1 (36)	3.9 (23)	1.4 (5)	
Use of Lipid lowering drugs	32.7 (920)	43.6 (721)	57 (1017)	72.7 (431)	77.3 (282)	0.001
Patients with Diabetes	24.4 (686)	28.8 (475)	17.8 (318)	34.7 (206)	20.3 (74)	
Duration of diabetes (years)	1.4 ± 0.09	1.8 ± 0.12	1.3 ± 0.12	2.2 ± 0.21	1.3 ± 0.26	
Use of oral anticoagulants	43.1 (1211)	51.7 (854)	60 (1071)	74.5 (442)	77.3 (282)	0.001
Endpoints						
*End stage renal failure*	35	14	5	2	1	
*Reno-atherosclerotic disease*	15	3	5	1	0	
*Number of total events*	50	17	10	3	1	
*Total person years*	14209	8195	7766	2205	1218	
*Incidence rate per 1000 population*	3.52	2.07	1.29	1.36	0.82	< 0.001

Continuous variables are expressed as age and sex adjusted means with SE; categorical variables are expressed as percentages with numbers in parenthesis; ^a^median with interquartile range

Among those with clinically manifest vascular disease, 29.2% achieved the systolic blood pressure treatment target, 43.3% the diastolic blood pressure target, 33.7% the total cholesterol target, 34.8% the LDL target and 78.9% the appropriate treatment target for albuminuria. Among the diabetic participants, 24.6% achieved the systolic blood pressure treatment target, 38.1% the diastolic blood pressure target, 33.9% the total cholesterol target, 38.2% the LDL target, 41% achieved appropriate treatment of albuminuria and 26.9% achieved the HbA1c target. The diabetics had poorer control of blood pressure and less use of RAS inhibitors for the treatment of albuminuria than the general cohort.

### Primary objective

At the end of the follow up period, there were 81 renal endpoints (Table 2). The unadjusted incidence rate for renal endpoints was 2.41 per 1,000 person-years. There were 57 renal endpoints for end stage renal failure with an incidence rate of 1.7 per 1,000 person-years. For renal atherosclerotic disease, there were 24 events with an incidence rate of 0.71 per 1,000 person-years. There was a statistically significant linear trend (p value less than 0.001) for incidence rate reduction with increasing target achievement.

The attainment of two or more treatment targets at baseline decreased the unadjusted risk of renal endpoint development for all groups except the diabetics (Table 3). The decreased unadjusted risks were statistically significant for trends. After adjustment for confounding variables such as age, sex, eGFR, albuminuria status, haemoglobin and hypertension, the hazard ratios for renal endpoints remained statistically significant for trends in the overall cohort and for those with clinically manifest vascular disease.

**Table 3:** Hazard ratios of renal endpoints by number of cardiovascular risk factor treatment targets achieved

	All			Clinically Manifest Vascular Disease			Diabetes		
	HR	(95% CI)	p for trends	HR	95% CI	p for trends	HR	95% CI	p for trends
n	7208			4859			1759		
number of events	81			52			28		
**Renal endpoint**									
Model 1 (crude)									
*Targets achieved *									
≤ 1	1.00		0.001	1.00		0.0001	1.00		0.704
2	0.59	(0.34, 1.02)		0.51	(0.26, 1.00)		0.69	(0.27, 1.81)	
3	0.36	(0.18, 0.71)		0.23	(0.09, 0.59)		1.09	(0.39, 3.05)	
4	0.37	(0.12, 1.19)		0.29	(0.07, 1.22)		0.99	(0.29, 3.47)	
≥ 5	0.22	(0.03, 1.59)		0	0		0	0	
									
Model 2 adjusted	a			b			c		
*Targets achieved *									
≤ 1	1.00		0.018	1.00		0.021	1.00		0.174
2	0.49	(0.28, 0.86)		0.58	(0.29, 1.14)		0.37	(0.13, 1.00)	
3	0.54	(0.27, 1.09)		0.38	(0.15, 0.99)		0.62	(0.22, 1.80)	
4	0.46	(0.14, 1.51)		0.64	(0.15, 2.69)		0.54	(0.15, 1.99)	
≥ 5	0.42	(0.06, 3.11)		0	0		0	0	
									
Model 3 adjusted ^d^									
*Targets achieved *									
≤ 1	1.00		0.013	1.00		0.018	1.00		0.107
2	0.46	(0.26, 0.82)		0.56	(0.28, 1.12)		0.28	(0.10, 0.79)	
3	0.51	(0.26, 1.03)		0.36	(0.14, 0.95)		0.39	(0.12, 1.26)	
4	0.49	(0.15, 1.60)		0.63	(0.15, 2.67)		0.56	(0.16, 2.01)	
≥ 5	0.37	(0.05, 2.76)		0	0		0	0	
									
**Risk factors **^e^									
SBP (per mmHg)	1.02	(1.01, 1.03)		1.02	(1.01, 1.03)		1.03	(1.01, 1.05)	
LDL (per mmol/L)	1.26	(1.04, 1.53)		1.31	(1.02, 1.68)		1.21	(0.83, 1.75)	
Treatment for albuminuria	0.90	(0.57, 1.43)		0.97	(0.55, 1.70)		0.58	(0.24, 1.39)	
HbA1c (per %)	1.18	(1.01, 1.38)		1.20	(0.96, 1.49)		1.44	(1.12, 1.84)	

^a^adjusted for hypertension, albuminuria, eGFR and haemoglobin levels

^b^adjusted for hypertension, albuminuria and eGFR levels

^c^adjusted for eGFR and haemoglobin levels

^d^adjusted for Model 2 + age and sex

^e^adjusted for age, sex, hypertension, albuminuria, eGFR and haemoglobin levels

### Secondary objective

When individual cardiovascular risk factors were compared, there was an increased risk of developing any renal endpoint for each increase in unit of systolic blood pressure, LDL and HbA1c levels. The hazard ratios for all risk factors were similar for all subgroups of patients, except for the hazard ratio of HbA1c among diabetics. Each unit increase in HbA1c levels suggests a higher rate of increase in the hazard ratio for renal complications in diabetics, than in the general cohort or in those with clinically manifest vascular disease.

## Discussion

In this cohort study, we studied whether the attainment of treatment targets established for the prevention of cardiovascular disease would concomitantly reduce the rate of renal complications. Based on the current 2007 European clinical practice guidelines, we observed that the achievement of any two or more treatment targets at baseline do reduce the rates of renal complications, for patients at high risk of atherosclerotic disease and for those with clinically manifest vascular disease. Although this observation was not statistically significant for diabetics, it may be clinically beneficial. A previous study of diabetics showed a decline in renal complications if three or more treatment targets were achieved [26]. With increasing treatment targets achieved, a lower haemoglobin concentration level was seen. However, it was not associated with poorer outcomes. This may be a result of more liberal use of RAS inhibitors, which decreases erythropoiesis [27]. There was no optimal number of cardiovascular risk factor treatment targets to achieve. The risk of renal complications decreased linearly as more targets were attained; consistent with the notion that no J-curve exists for renal outcomes.

There are distinct differences between the diabetic and clinically manifest vascular disease population cohorts. Primarily, the decreased hazard ratios for renal outcomes in diabetics were only observed after the model was adjusted for renal confounders such as eGFR and haemoglobin levels. This may be because once chronic renal disease has set in, achievement of treatment targets alone is insufficient to reduce or retard the rate of complications in diabetics. This was not the case for those with manifest vascular disease, where there was a consistent reduced risk of renal complications even with non-adjustment for renal confounders. The second distinct difference is, there does not appear to be a gradient decreased hazard ratio for diabetics as the number of treatment targets achieved increased. A previous study of treatment targets and cardiovascular outcomes in diabetics showed a graded response [28].

The low percentage of participants achieving treatment targets is consistent with other countries. In a Swedish study, only 17% of their hypertensive population had achieved a systolic blood pressure target of less than 140 mmHg and 64% achieved the diastolic blood pressure target of less than 90 mmHg [29]. In the Losartan Intervention For Endpoint reduction in hypertension (LIFE) study, which was a multi country randomized control trial, only 16.3% had achieved the total cholesterol target of less than 5.0 mmol/l at baseline [30]. In the United Kingdom, only 19.7% and 26.9% achieved blood pressure and cholesterol targets respectively [31]. More patients achieving the target for diastolic blood pressure than for systolic blood pressure is also consistent with other studies [29]. This is probably reflective of older, stiffer arteries which is expected in high risk patients, and not because therapies used were more effective in lowering diastolic blood pressure.

Looking at the individual cardiovascular risk factor levels, the findings amongst the study population is consistent with current literature. With increasing systolic blood pressure [32], LDL and HbA1c, the risk for renal complications increases statistically significantly. Considering the similar risks for renal complications in all cohort groups with increasing systolic blood pressure and LDL levels, the treatment of HbA1c levels is crucial in diabetics. The non-significant decreased hazard ratio seen in all patient groups for the treatment of albuminuria may be due to two reasons; suboptimal dosing or discontinuation of medication due to non-response to blood pressure lowering with ACEI or ARB use. Inadequate dosing of ACEI or ARB's causes less anti-albuminuric activity thus there is suboptimal reduction in albuminuria. Aside from this, non-response to blood pressure reduction does not mitigate the effects of anti-albuminuric activity; therefore discontinuation of these drugs increases the risk of renal endpoints [33].

This study has a few limitations. The number of treatment targets achieved was determined only at baseline. Over the entire duration of follow up, patients may fluctuate between different categories of targets achieved, depending on clinical management received.

The duration of follow up may be insufficient for ESRF development in many patients. In the UKPDS study, development of renal endpoints occurred in only 0.8% after a median of 10 years [34]. Aside from this, the number of events that occurred was small, 81 in total and in diabetics 28. This may explain the lack of power in proving statistical significance, except for the tests for trends. A longer follow up duration would provide more accurate estimates and therefore stronger evidence. Nevertheless, this study had a total sample size of 7,208 participants with a sum of 33, 593 person-years of follow up. This is sizable.

Another key strength of this study is the use of hard endpoints; end stage renal failure and symptomatic renal vascular disease requiring intervention. We did not use surrogate endpoints such as rate of GFR decline because in diabetics and in other patient populations, the calculated GFR using the MDRD formula underestimates the rate of renal function loss [35-37]. It also becomes inaccurate as renal function improves; real GFR can fluctuate between 35 to 90 mL min^-1 ^1.73 m^-2 ^for an eGFR of 60 mL min^-1 ^1.73 m^-2 ^[38]. Aside from this, stabilization or regression of renal function decline has been known to occur in patients treated with ACEI despite having severe GFRs [39]. In this study, the maximum survival time for an eGFR of less than 15 mL min^-1 ^1.73 m^-2 ^was 6.27 years.

These hard endpoints reflect different pathophysiological mechanisms. End stage renal failure may be caused by both micro and macrovascular changes whereas renal vascular disease is caused by macrovascular changes. Despite these differing pathophysiological mechanisms, these findings are suggestive that the same treatment targets reduce their risk of development.

## Conclusion

This study shows that current clinical practice guideline targets for the prevention of cardiovascular disease are useful in concomitantly reducing the risk of renal complications; in any patient population at high risk for vascular diseases. If more patients can be adequately treated to achieve the cardiovascular risk factor treatment targets, there will be important reductions in renal complications.

## Competing interests

The authors declare that they have no competing interests.

## Authors' contributions

SS conceived the research idea, performed the statistical analysis and drafted the manuscript.

YVDG designed and coordinated the study and helped draft the manuscript.

FLJV participated in the design and coordination of the study, and helped draft the manuscript.

MLB participated in the conception of the idea, analysis and helped draft the manuscript.

All the authors have read the final manuscript and have given their approval for it to be presented in its present form.

## SMART Study Group

Members of the SMART Study Group are Ale Algra, MD, PhD, Pieter A. Doevendans, MD, PhD, Yolanda van der Graaf, MD, PhD, Diederick E. Grobbee, MD, PhD, L. Jaap Kappelle, MD, PhD, Willem P. Th. M. Mali, MD, PhD, Frans L. Moll, MD, PhD, Guy E.H.M. Rutten, MD, PhD, and Frank L. J. Visseren, MD, PhD.

## Pre-publication history

The pre-publication history for this paper can be accessed here:

http://www.biomedcentral.com/1471-2261/11/40/prepub
